# Anatomical and Physiological Changes after Paclitaxel-Coated Balloon for Atherosclerotic *De Novo* Coronary Lesions: Serial IVUS-VH and FFR Study

**DOI:** 10.1371/journal.pone.0147057

**Published:** 2016-01-29

**Authors:** Soe Hee Ann, Gillian Balbir Singh, Kyung Hun Lim, Bon-Kwon Koo, Eun-Seok Shin

**Affiliations:** 1 Department of Cardiology, Ulsan University Hospital, University of Ulsan College of Medicine, Ulsan, South Korea; 2 Department of Internal Medicine and Cardiovascular Center, Seoul National University Hospital, Seoul, South Korea; Cliniche Humanitas Gavazzeni, ITALY

## Abstract

**Aims:**

To assess the serial changes of *de novo* coronary lesions treated with paclitaxel-coated balloon (PCB) using intravascular ultrasound virtual histology (IVUS-VH) and fractional flow reserve (FFR).

**Method and Results:**

This prospective observational study enrolled 27 patients with coronary artery disease treated with PCB who underwent coronary angiography, IVUS-VH and FFR before, immediately after intervention and at 9 months. 28 *de novo* lesions were successfully treated with PCB. Angiographic late luminal loss was 0.02 ± 0.27mm. Mean vessel and lumen areas showed increase at 9 months (12.0 ± 3.5mm^2^ to 13.2 ± 3.9mm^2^, p <0.001; and 5.4 ± 1.2mm^2^ to 6.5 ± 1.8mm^2^, p <0.001, respectively). Although mean plaque area was unchanged (6.6 ± 2.6mm^2^ to 6.6 ± 2.4mm^2^, p = 0.269), percent atheroma volume decreased significantly (53.4 ± 7.9% to 49.5 ± 6.4%, p = 0.002). The proportion of plaque compositions including fibrous, fibrofatty, dense calcium and necrotic core by IVUS-VH was unchanged at 9 months. The FFR of the treated lesion was 0.71 ± 0.13 pre-procedure, 0.87 ± 0.06 post-procedure and 0.84 ± 0.06 at follow-up.

**Conclusions:**

*De novo* coronary lesions treated with PCB showed persistent anatomical and physiological patency with plaque redistribution and vessel remodeling without chronic elastic recoil or plaque compositional change during follow-up.

## Introduction

Non-stent based local drug delivery using paclitaxel-coated balloons (PCB) has emerged as a new clinical treatment alternative by maintaining the anti-proliferative properties of drug-eluting stents (DES).[1] As the caged vessel after metal stent implantation excludes late lumen enlargement and vascular remodelling, PCB might have an additional benefit.[2] In a recent study, small vessel de novo lesions treated with PCB in unselected patients showed low rates of target lesion revascularization and major adverse cardiac events. PCB was suggested as an alternative treatment option to drug eluting stents[3].[4] However, despite the effectiveness of PCB in de novo lesion, anatomical and physiological responses after PCB have not been fully explored. The aim of our study was to assess the serial changes of de novo lesions treated with PCB. We performed serial angiographic, fractional flow reserve (FFR), and intravascular ultrasound virtual histology (IVUS-VH) measurements before and immediately after intervention, and at 9 months follow-up in de novo lesions treated with PCB.

## Methods

### Study population

This study is a prospective, observational, single-arm study aimed at assessing functional and intravascular morphological changes induced by the PCB in de novo lesions. This study was carried out according to the guidelines of the Declaration of Helsinki and was approved by the Institutional Review Board ethics committee at Ulsan University Hospital. All patients in this study provided signed informed consent.

### Patient selection

Patients with stable or unstable angina pectoris, with de novo coronary lesions scheduled to undergo percutaneous coronary intervention were considered eligible for this study. The inclusion criteria were lesions with a reference vessel diameter between 2.5mm and 3.5mm and a lesion length of ≤24mm. Exclusion criteria were left ventricular ejection fraction <30%, acute myocardial infarction, left main disease, ostial lesion, heavily calcified or thrombotic lesion, life expectancy <1 year and known renal failure (creatinine >2 mg/dl).

### Interventional procedure, device, FFR data acquisition and analysis

All patients were treated with acetylsalicylic acid 200mg and clopidogrel 300 to 600mg loading dose before the procedure and 100U/Kg of unfractionated heparin was injected intravenously to maintain an activated clotting time ≥250s during the procedure. For the lesion preparation, the patient underwent pre-dilation with an optimally sized balloon based on angiography (1:1 balloon-to-vessel ratio), shorter than the intended length of PCB with nominal pressure inflation of 8–14 atm. FFR far distal to the lesion site was acquired before and after balloon angioplasty. Based on the FFR value after balloon angioplasty, if plain old balloon angioplasty (POBA)-FFR was favorable, PCB was selected primarily by operators. Under angiographic guidance, PCB (SeQuent Please^®^, paclitaxel-coated balloon catheter, B. Braun, Melsungen, Germany) sized at 1:1 balloon-to-vessel ratio, was delivered rapidly (median of 15 seconds) and inflated for 60 seconds with nominal pressure (8 to 12 atm). The use of glycoprotein IIb/IIIa inhibitors during the procedure was at the discretion of the operator. Device success was defined as angiographic success (final residual stenosis <30% by visual estimate and Thrombolysis in Myocardial Infarction flow grade 3), using the PCB device. Coronary angiographies were analyzed using the Cardiovascular Angiography Analysis System (CAAS 5.10, Pie Medical Imaging B.V., Maastricht, The Netherlands) by an independent investigator before the procedure, after PCB application and at 9 months follow- up.

### IVUS-VH acquisition and analysis

200μg intracoronary nitroglycerin was administered routinely before the image acquisition at baseline and at repeat assessment at 9 months follow-up. Baseline IVUS-VH assessments were performed before and after balloon angioplasty. Using the motorized transducer pullback system (0.5 mm/s), the 2.9-F IVUS imaging catheter (Eagle Eye, Volcano Corp., Rancho Cordova, California) incorporated a 20-MHz phased-array transducer. Offline analyses were done with the computer VH software program (pcVH 2.2; Volcano Therapeutics, Rancho Cordova, California, USA) by an independent examiner who was unaware of the clinical characteristics of the participants. Geometric and compositional characteristics of the de novo lesions treated with PCB were analyzed, and lesions were confirmed with side by side angiography of distinguished branches. Geometric quantitative IVUS analyses were performed according to criteria from the IVUS clinical expert consensus document.[5] External elastic membrane (EEM), lumen, and plaque & media (P&M) (P&M = EEM—lumen) areas were obtained by manual contour detection. Percent atheroma volume was calculated as the proportion of the entire vessel wall occupied by atherosclerotic plaque, throughout the segment of interest. We used this equation; Percent atheroma volume = Σ P&M area/ Σ EEM area X100.[6]

Compositional tissue characteristic areas were expressed in colors, as previously described (green for fibrous, yellow-green for fibrofatty, white for dense calcium and red for necrotic core).[7] The IVUS-VH analyses were reported as mean area of each composition and as percentage change of volume. Percentage change (%) of all areas was calculated as follows: ([follow-up value–baseline value] / [baseline value] x 100). Plaque phenotype was identified based on plaque composition as pathologic intimal thickening, IVUS-VH derived thin-cap fibroatheroma, thick-cap fibroatheroma, fibrotic plaque and fibrocalcific plaque by two experienced, independent investigators.[8,9]

### Follow-up and endpoints

All patients were scheduled to undergo clinical and angiographic follow-up at 9 months. The primary endpoints of this study were serial changes in IVUS-VH and FFR parameters. Secondary endpoints included procedural success; angiographic measurements; and clinical outcomes according to the Academic Research Consortium criteria [10]. Procedural success was defined as angiographic success without the occurrence of in-hospital major adverse cardiac events.

### Statistical analysis

All statistical analyses were performed using SPSS (Version 18.0; SPSS Inc, Chicago, Illinois). Categorical variables are presented as frequencies with percentage and continuous variables are presented as mean ± standard deviation. Data at different time points were analyzed with the paired Student’s *t* test. Categorical variables were compared using the chi-square or Fisher exact test, as appropriate. A two sided P-value of less than 0.05 was used to indicate statistical significance.

## Results

### Clinical and procedural characteristics

Among forty-four patients (45 lesions) treated with PCB, twenty-seven patients (28 lesions) who underwent IVUS-VH and FFR measurement were included in this study. Table 1 shows the baseline and clinical characteristics of the participants. Mean age was 59.4 ± 6.5 years and 18 patients were male (64.3%). Seventeen patients (63.0%) were presented as stable angina, 10 patients (37.0%), as unstable angina. The most commonly affected epicardial artery was the left anterior descending artery, 17 lesions (60.7%). The size of applied PCB catheter and maximal inflation pressure are shown in Table 1. There was no in-hospital clinical event including peri-procedural myocardial infarction and the procedural success rate was 100%. At discharge, all patients received statins; 24 were on 20mg of atorvastatin and 3 were on 10mg of rosuvastatin. Other medications are listed in Table 1 including the use of 6 weeks of clopidogrel and aspirin. At the 9 months follow-up, there was significant decrease in the total cholesterol, triglyceride and low density lipoprotein (LDL)-cholesterol levels compared to baseline. However, the high density lipoprotein (HDL)-cholesterol and high sensitivity C-reactive protein (hsCRP) levels showed no significant changes from baseline to 9 months (Table 2).

**Table 1:** Baseline and procedural characteristics.

Variables	(n = 27)
Age (years)	59.4 ± 6.5
Male (%)	18 (64.3)
**Cardiovascular risk factors**	
Diabetes	7 (25.0)
Hypertension	15 (53.6)
Current smoker	9 (32.1)
Hypercholesterolemia	13 (46.4)
Family History of CAD	4 (14.3)
**Clinical manifestation**	
Stable angina	17 (63.0)
Unstable angina	10 (37.0)
Medications at discharge	
Aspirin	27 (100)
Clopidogrel	27 (100)
ACEi/ARB	9 (33.3)
B-blocker	7 (25.9)
Calcium channel blocker	13 (48.1)
Statin	27 (100)
Atorvastatin	24 (88.9)
Rosuvastatin	3 (11.1)
Angiographic findings of target lesion	(n = 28)
**Coronary artery, n (%)**	
Left anterior descending	17 (60.7)
Left circumflex	5 (17.9)
Right coronary	6 (21.4)
**Lesion type (B2 & C)**	19 (67.8)
Paclitaxel-Coated Balloon	
Device diameter (mm)	3.00 ± 0.35
Device length (mm)	23.1 ± 4.4
Device pressure (atm)	9.1 ± 1.8
Inflated device diameter (mm)	3.11 ± 0.37

Values are presented as numbers (%) or mean ± standard deviation.

CAD, coronary artery disease; ACEi, angiotensin converting enzyme inhibitor; ARB, angiotensin receptor blocker; B-blocker, Beta adrenergic receptor blocker.

**Table 2:** Laboratory results.

	Baseline	9 months Follow-up	Change (%)	p
Body mass index	24.2 ± 2.1	24.3 ± 2.0	1.0 ± 9.3	0.703
Total Cholesterol (mg/dL)	181.0 ± 40.0	135.2 ± 19.3	-22.1 ± 19.5	<0.001
Triglyceride (mg/dL)	154.3 ± 81.6	105.0 ± 44.4	-22.1 ± 34.2	0.002
HDL-Cholesterol (mg/dL)	47.7 ± 9.3	46.4 ± 9.3	-1.2 ± 16.9	0.435
LDL-Cholesterol (mg/dL)	103.1 ± 27.4	65.1 ± 13.5	-32.5 ± 22.9	<0.001
hsCRP (mg/L)	0.13 ± 0.18	0.08 ± 0.15	-7.4 ± 91.8	0.075

Values are presented as mean ± standard deviation.

HDL, high-density lipoprotein; LDL, low-density lipoprotein; hsCRP, high sensitivity C-reactive protein.

### Angiographic follow-up and adverse events at 9 months

All patients underwent clinical and angiographic follow-up at 9 months. The minimal luminal diameter and percentage diameter stenosis of target lesions by Quantitative Coronary Angiography were 1.04 ± 0.46 mm and 59.6 ± 14.4%, respectively (Table 3). After PCB application, minimal lumen diameter increased to 2.02 ± 0.40 mm and diameter stenosis decreased to 25.5 ± 8.6%. Angiographic follow-up at 9 months showed similar minimal lumen diameter and diameter stenosis and the late luminal loss was 0.02 ± 0.27 mm. There were no angiographic binary restenosis or cardiovascular events during the 9 months follow-up except 1 case of non-target lesion progression in a treated vessel with PCB (median follow-up duration: 276 days).

**Table 3:** Quantitative coronary angiography and functional measurements.

	Pre-balloon	Post-balloon	9 months follow-up
**Quantitative Coronary Angiography**	**n = 28**	**n = 28**	**n = 28**
Reference vessel diameter, mm	2.53 ± 0.46	2.71 ± 0.45	2.58 ± 0.44
Minimal lumen diameter, mm	1.04 ± 0.46	2.02 ± 0.40	2.01 ± 0.53
Diameter stenosis, %	59.6 ± 14.4	25.5 ± 8.6	25.3 ± 11.7
Lesion length, mm	21.3 ± 5.4	22.5 ± 5.0	21.7 ± 5.1
Acute gain, mm	-	0.92 ± 0.46	-
Late-luminal loss, mm	-	-	0.02 ± 0.27
Net gain, mm	-	-	0.89 ± 0.53
Binary restenosis	-	-	0
**FFR**	**n = 22**	**n = 28**	**n = 25**
	0.71 ± 0.13	0.87 ± 0.06	0.84 ± 0.06

Values are presented as mean ± standard deviation.

PCB, paclitaxel-coated balloon; FFR, fractional flow reserve.

### Serial FFR and IVUS-VH changes

**Fig 1:**
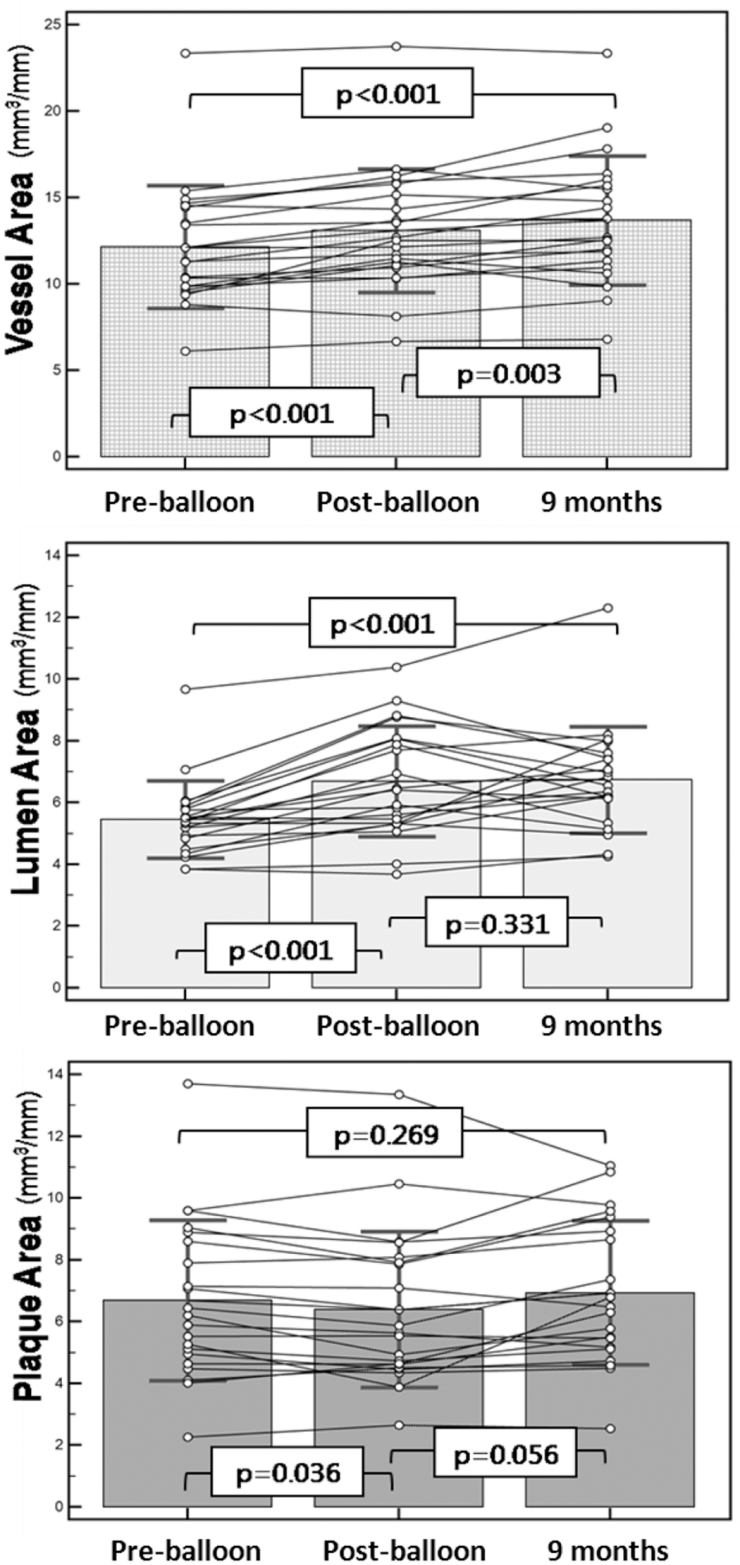
Serial changes in mean vessel, lumen and plaque area.

**Fig 2:**
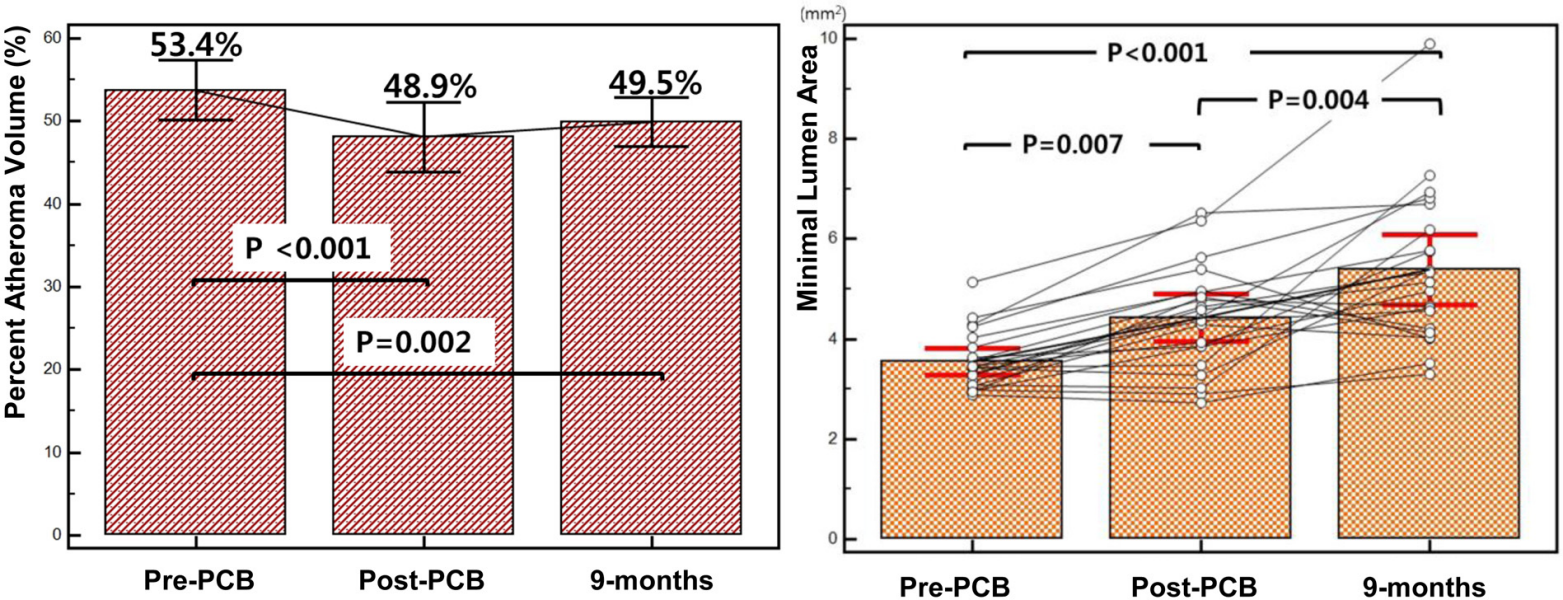
Serial changes in percent atheroma volume and minimal lumen areas.

**Fig 3:**
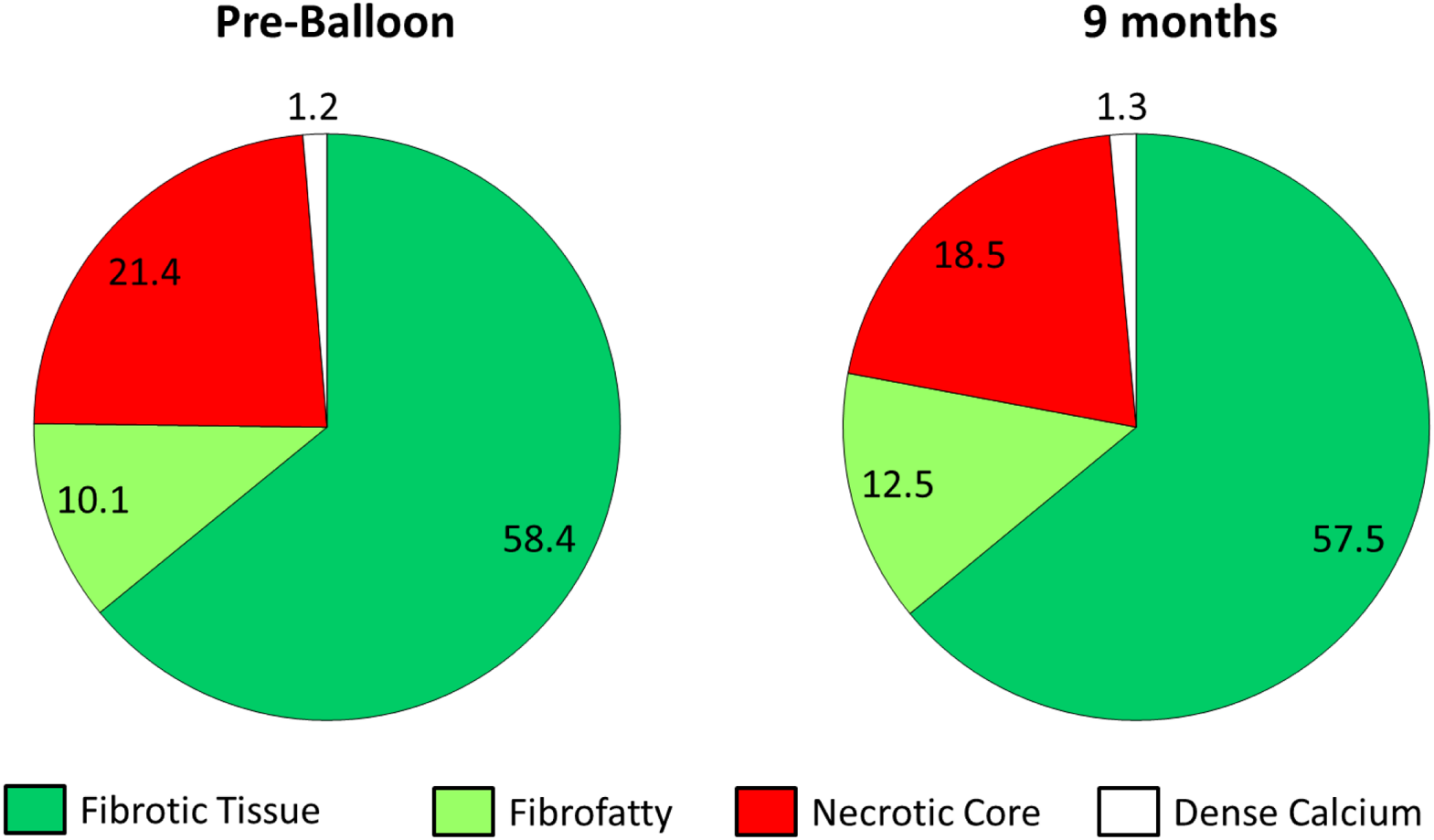
Serial changes in plaque composition.

**Fig 4:**
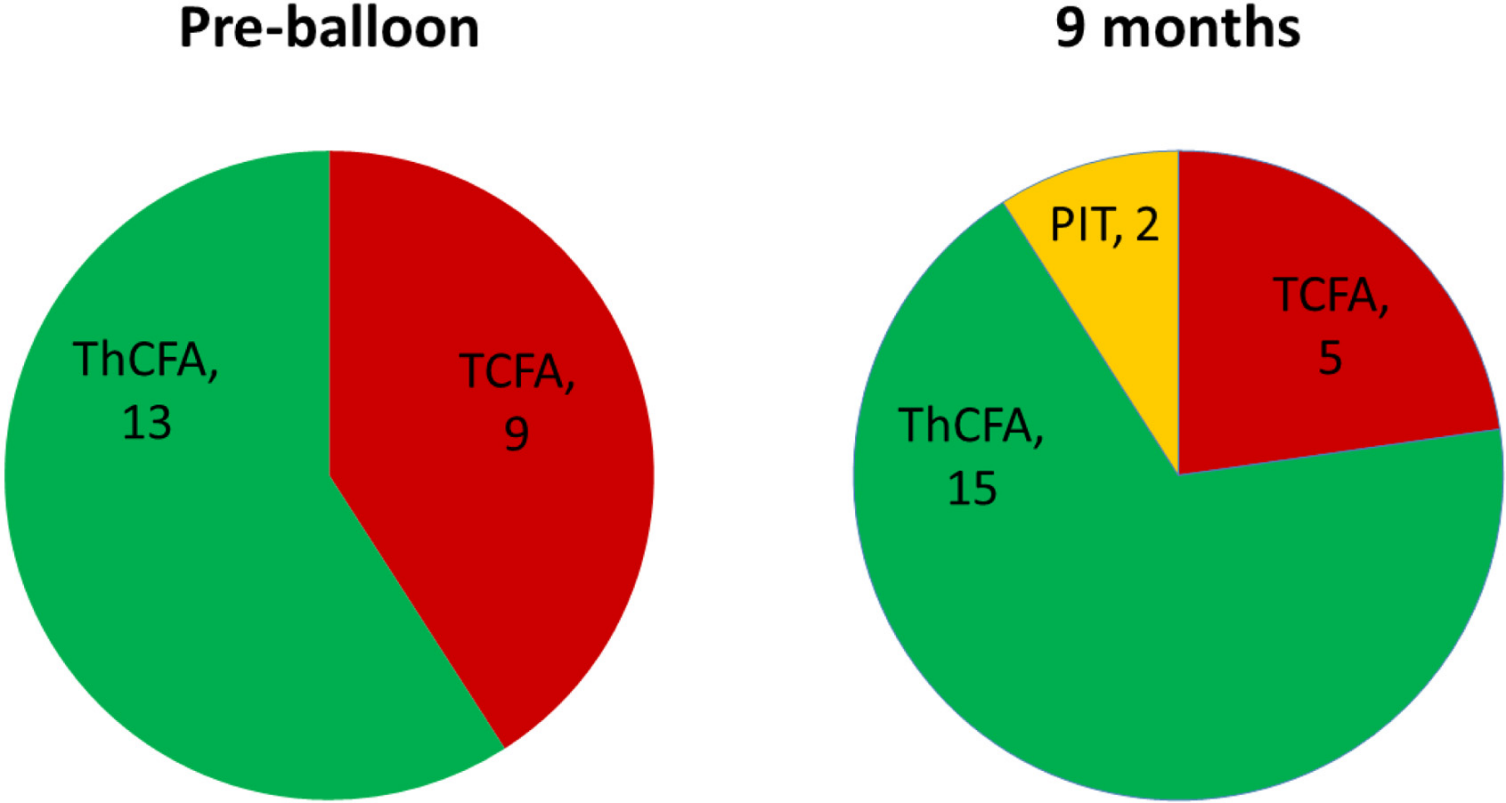
Serial changes of plaque phenotypes. TCFA = thin-cap fibroatheroma, ThCFA = thick-cap fibroatheroma, PIT = pathologic intima thickening.

**Fig 5:**
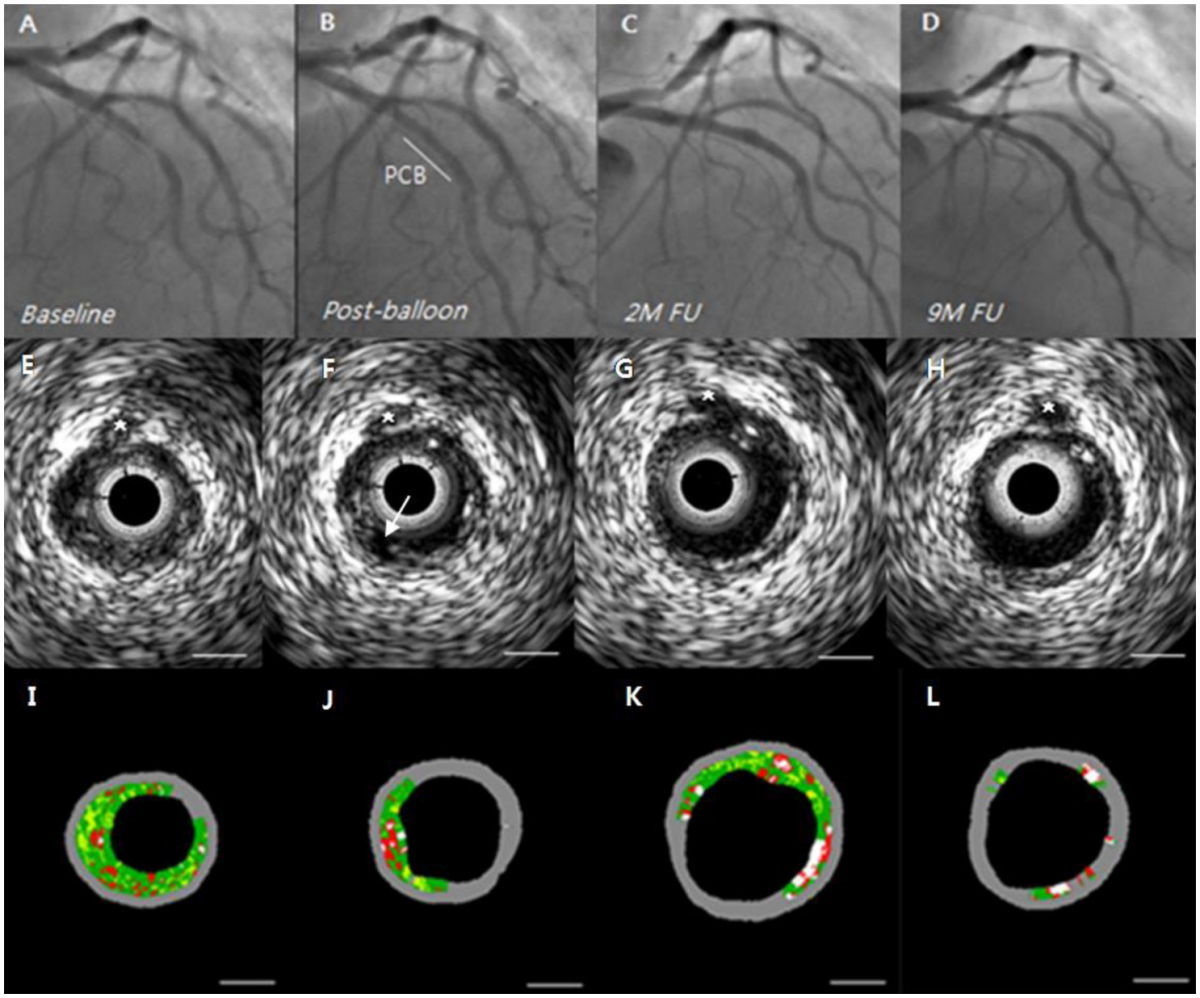
A representative case of paclitaxel-coated balloon treatment in a de novo lesion. A 67-year-old woman who had unstable angina with near total occlusion of the left anterior descending coronary artery (A) underwent paclitaxel-coated balloon (PCB) treatment after plain balloon angioplasty (POBA). After POBA, the lesion showed minimal residual stenosis including a non-flow-limiting type A dissection (B). The patient experienced no procedural-related complications and was discharged without symptoms after the procedure. She took dual antiplatelet therapy for 6 weeks followed by aspirin alone. Serial intravascular ultrasound virtual histology was performed right after POBA and at 2 months and 9 months follow-up. After POBA, the lesion was well expanded with dissection of plaque seen on IVUS (F). At 2 months, IVUS demonstrated an enlarged lumen accompanied by a slightly enlarged vessel (G). At 9 months, the lumen and vessel were both well preserved (H). The plaque phenotype, fibroatheroma remain unchanged and the plaque burden (PB) decreased over time (I; 66%, J; 50%, K; 47%, L: 40%). Plaque burden (%) was defined as P&M area divided by EEM area x 100. Asterisks and arrows indicate a small septal branch and a dissected flap, respectively. Bar = 1 mm.

After balloon angioplasty, mean FFR value improved from 0.71 ± 0.13 to 0.87 ± 0.06 (p <0.001). At 9 months follow-up, there was no change in FFR value (0.84 ± 0.06, p = 0.326) (Table 3). Geometric and compositional changes are shown in Tables 4 and 5. Overall, there were significant changes in mean vessel area (EEM) & lumen area (Fig 1 and S1 File). Mean vessel area increased from 12.0 ± 3.5mm^2^ to 13.2 ± 3.9mm^2^ (p <0.001), and lumen area also increased from 5.4 ± 1.2mm^2^ to 6.5 ± 1.8mm^2^ (p <0.001). Mean plaque area however, was unchanged (6.6 ± 2.6mm^2^ to 6.6 ± 2.4mm^2^, p = 0.269). Percent atheroma volume decreased significantly (53.4 ± 7.9% to 49.5 ± 6.4%, p = 0.002) and minimal lumen area showed a corresponding increase after balloon angioplasty and continued to increase at 9 months (4.22 ± 1.02mm^2^ to 5.21 ± 1.41mm^2^, p = 0.004) (Fig 2). There were no aneurysmal changes detected on IVUS. All four IVUS-VH plaque compositions were unchanged during the follow-up (Fig 3). However, among the 9 IVUS-VH derived thin-cap fibroatheroma detected at baseline, only 5 remained at follow-up; 2 converting to thick-cap fibroatheroma and 2 to pathologic intima thickening (Fig 4). A representative case of serial IVUS-VH is presented in Fig 5.

**Table 4:** Serial changes of grey-scale IVUS parameters in pre- and post-balloon and at 9 months follow-up.

	Pre-balloon (n = 22)	Post-balloon (n = 27)	9 months (n = 28)	p value		
Mean area, mm^2^				pre-balloon vs. post-balloon	post-balloon vs. 9 months	pre-balloon vs. 9 months
EEM	12.0±3.5	12.7±3.9	13.2±3.9	**<0.001**	**0.003**	**<0.001**
Lumen	5.4±1.2	6.3±1.9	6.5±1.8	**<0.001**	0.331	**<0.001**
Plaque	6.6±2.6	6.3±2.6	6.6±2.4	**0.036**	0.056	0.269
Percent atheroma volume (%)	53.4±7.9	48.9±9.0	49.5±6.4	**<0.001**	0.623	**0.002**
Minimal lumen area (mm^2^)	3.52±0.52	4.22±1.02	5.21±1.41	**0.007**	**0.004**	**<0.001**

Values are presented as mean ± standard deviation. Paired t-test was used. EEM, external elastic membrane.

**Table 5:** Serial changes of IVUS-VH parameters in pre- and post-balloon and at 9 months follow-up.

	Pre-balloon (n = 22)	Post-balloon (n = 27)	9 months (n = 28)	p value		
Mean area, mm^2^				pre-balloon vs. post-balloon	post-balloon vs. 9 months	pre-balloon vs. 9 months
Necrotic core	0.76±0.60	0.69±0.58	0.61±0.46	0.68	0.467	0.153
Dense calcium	0.46±0.43	0.44±0.31	0.43±0.38	0.794	0.821	0.952
Fibro-fatty	0.32±0.17	0.27±0.23	0.42±0.34	**0.002**	**0.038**	0.103
Fibrous tissue	2.11±1.23	1.81±1.41	2.01±1.24	**<0.001**	0.154	0.955

Values are presented as mean ± standard deviation.

## Discussion

The main findings of this prospective observational study of de novo coronary lesions treated with PCB are: 1) mean vessel area and lumen area significantly increased after 9 months; 2) mean plaque area remained unchanged, however percent atheroma volume decreased significantly; 3) all four IVUS-VH plaque compositions were unchanged; and 4) the functional patency was maintained during the 9 months follow-up.

Plain balloon angioplasty was originally developed as a revascularization therapy which restores the coronary flow by intentional plaque modification.[11] However, elastic recoil and restenosis were major limitations.[12] In contrast, PCB was developed to deliver a single dose of paclitaxel by one minute of PCB inflation, which was proven in a preclinical trial.[1] As the main effects of PCB rely on the rapid transfer of an antiproliferative agent to the vessel wall, paclitaxel was adopted for use in drug coated balloon with prolonged tissue retention rates.[13] Paclitaxel exerts potent antiproliferative effects by binding to the subunit of tubulin, resulting in arrest of microtubule function, promoting prolonged antiproliferation.[14] As a result, paclitaxel can inhibit arterial smooth muscle cell proliferation and migration after being used locally.[15] Several randomized clinical trials have shown better angiographic outcomes of PCB treatment not only in in-stent restenosis compared to POBA[16] or DES; [17] but also in small vessel disease compared to DES.[18] Despite promising angiographic and clinical outcomes, there is limited data on PCB treatment in de novo lesions of coronary arteries because of the concern of acute recoil and abrupt closure directly after the procedure. However, as the main pathophysiology of restenosis after POBA is arterial remodeling rather than neointimal hyperplasia, it may be helpful to investigate the mechanism of vascular response after PCB treatment. According to the previous serial IVUS study [19] on 212 lesions after balloon angioplasty, 73% of the decrease in lumen area was due to a decrease in vessel area (EEM) and 27% was due to an increase in plaque area. Unlike these results seen with POBA, [20,21] plaque was not increased and vessel and lumen size enlarged after 9 months suggesting that both intimal hyperplasia and arterial constriction were prevented with coated paclitaxel use in this study. Although this advantageous arterial remodeling was reported as a case report using IVUS,[2] the present study raises a clinically valuable point in vascular response for the first time, which can explain the good angiographic outcome as well as the maintained coronary physiology. The majority of plaque composition was fibrous, followed by necrotic core and fibro-fatty tissue. Although there was no significant change in percentage of plaque compositions, fibrofatty tissue showed an increasing trend from baseline to 9 months follow-up.

Low-density lipoprotein cholesterol reduction after statin treatment has been reported to induce regression of plaque volume. [22] However, plaque volumes did not decrease after 9 months. In this study, moderate intensity statin therapy was used and the mean reduction of 32.5 ± 22.9% in baseline low-density lipoprotein cholesterol was inadequate to decrease plaque volumes. This might be insufficient compared to the recent guideline which recommends a reduction of > 50% of baseline LDL-cholesterol in clinical atherosclerotic coronary artery disease. [23] Although there may be concern regarding the late catch-up phenomenon from PCB treatment, this has not been thoroughly investigated as yet.[24] Therefore, high intensity statin treatment after PCB treatment may be beneficial in preventing late restenosis from progressive atherosclerosis.

It is encouraging that four VH-derived thin-cap fibroatheromas seen in this study converted to thick cap fibroatheroma or pathologic intima thickening at 9 months follow-up (Fig 4). Although the numbers were small, these findings suggest that plaque stabilization occurs with PCB.

### Study limitations

Firstly, selection bias may have occurred in individual cases. Only elective patients with a clinical diagnosis of stable or unstable angina and successful PCB application were included in the study mainly due to the complex protocol (i.e., pre- and post-procedural IVUS-VH and FFR). Secondly, although clinical and angiographic outcomes are promising, the nature of this registry does not allow for comparison with a reference technique. To establish the effects of PCB, a prospective randomized trial is needed to compare the changes in vascular response and plaque composition between plain balloon angioplasty and PCB treatment.

## Conclusions

De novo coronary lesions treated with PCB showed persistent patency with plaque redistribution without chronic elastic recoil and restored coronary blood flow resulting in increased lumen areas at follow-up.

## Supporting Information

S1 File Raw data of clinical and intravascular ultrasound. https://figshare.com/s/ea82a726fb714d99bd40.
(XLS)

## Abbreviations

DES: Drug eluting stent
EEM: External elastic membrane
FFR: Fractional flow reserve
HDL: High-density lipoprotein
hsCRP: high sensitivity C-reactive protein
IVUS: Intravascular ultrasound
IVUS-VH: Intravascular ultrasound virtual histology
LDL: Low density lipoprotein
PCB: Paclitaxel-coated balloon
POBA: Plain old balloon angioplasty
P&M: Plaque & Media
